# Cyclone-induced rapid creation of extreme Antarctic sea ice conditions

**DOI:** 10.1038/srep05317

**Published:** 2014-06-17

**Authors:** Zhaomin Wang, John Turner, Bo Sun, Bingrui Li, Chengyan Liu

**Affiliations:** 1 Polar Climate System and Global Change Laboratory, Nanjing University of Information Science and Technology (Nanjing Institute of Meteorology), Nanjing, China, 210044; 2 Earth System Modelling Center (ESMC), Nanjing International Academy of Meteorological Sciences(NIAMS), Nanjing University of Information Science and Technology, Nanjing, China, 210044; 3 British Antarctic Survey, High Cross, Madingley Road, Cambeidge CB3 0ET, UK; 4 Polar Research Institute of China, No. 451, Jinqiao Road, Pudong District, Shanghai, 200136, China

## Abstract

Two polar vessels, Akademik Shokalskiy and Xuelong, were trapped by thick sea ice in the Antarctic coastal region just to the west of 144°E and between 66.5°S and 67°S in late December 2013. This event demonstrated the rapid establishment of extreme Antarctic sea ice conditions on synoptic time scales. The event was associated with cyclones that developed at lower latitudes. Near the event site, cyclone-enhanced strong southeasterly katabatic winds drove large westward drifts of ice floes. In addition, the cyclones also gave southward ice drift. The arrival and grounding of Iceberg B9B in Commonwealth Bay in March 2011 led to the growth of fast ice around it, forming a northward protruding barrier. This barrier blocked the westward ice drift and hence aided sea ice consolidation on its eastern side. Similar cyclone-induced events have occurred at this site in the past after the grounding of Iceberg B9B. Future events may be predictable on synoptic time scales, if cyclone-induced strong wind events can be predicted.

Sea ice movement is strongly affected by the atmospheric circulation (e.g., ref. 1). A large and persistent atmospheric circulation anomaly over the Weddell Sea during the Austral summer of 2001/02 resulted in extreme sea ice conditions off the coast of Dronning Maud Land that stopped Halley Research Station being resupplied for a whole summer season^2^. On synoptic time scales, winds associated with cyclones can be very intense, particularly in the coastal regions of East Antarctic as cyclones often enhance strong katabatic wind events^3^,^4^. These strong winds can impose large atmospheric and subsequently induced oceanic forcing on sea ice, with great potentials to cause rapid establishment of extreme sea ice conditions on synoptic time scale. Around the Antarctic, ships have to navigate to re-supply the bases from early summer when such conditions can be created on synoptic time scale, thus making the polar navigation difficult.

In late December 2013, two polar vessels, Akademik Shokalskiy (ice strengthened) and Xuelong (an ice breaker), were trapped by thick sea ice in the Dumont d'Urville Sea (north of the coast of Adélie Land, East Antarctica), just to the west of 144°E and between 66.5°S and 67°S. This extreme event, which has attracted wide press and public attention across the world, demonstrated that thick sea ice can be created on synoptic time scales at this specific site. The ice was too thick (typically 2.5 m thick; Mingguang Li, personal communication) for Xuelong to navigate, as Xuelong can only break sea ice with a thickness of less than 1.2 m. This event offers an important opportunity to investigate how strong winds associated with cyclones force Antarctic sea ice change and even lead to the rapid creation of extreme sea ice conditions in the Antarctic coastal region.

## Results

**Figure 1:**
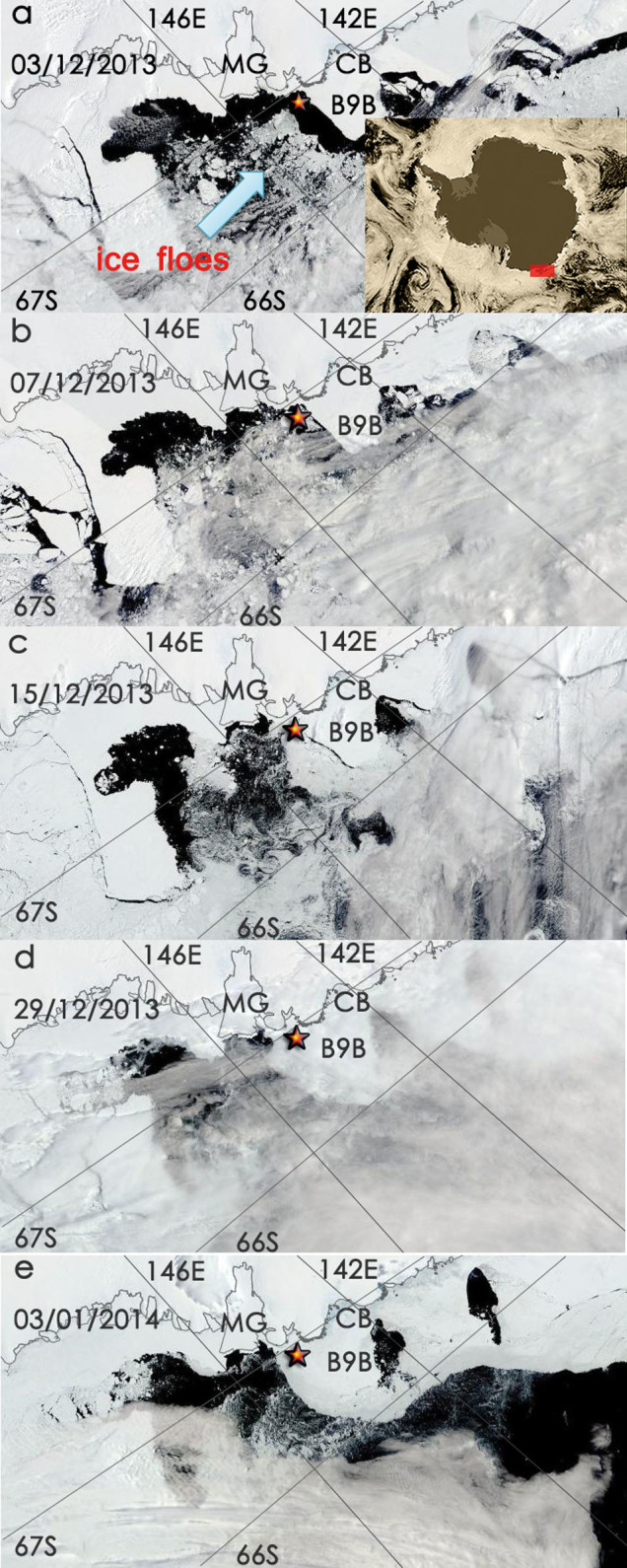
MODIS sea ice images for (a) 3 December 2013, (b) 7 December 2013, (c) 15 December 2013, (d) 29 December 2013, and (e) 3 January 2014. Mertz Glacier (MG), Commonwealth Bay (CB), and Iceberg B9B (B9B) are marked at the corresponding locations. Red star marks the site around which the two polar vessels were trapped. The red box in the inset in (a) shows the location of the interested region. The original images were captured from https://earthdata.nasa.gov/labs/worldview/, and were modified using Adobe Photoshop.

Sea ice distribution from high resolution MODIS (The Moderate Resolution Imaging Spectroradiometer) imagery (https://earthdata.nasa.gov/labs/worldview/) around the site where the two vessels were trapped in late December 2013 (hereafter termed “the site”) was characterized by ice fixed to the coast (fast ice) connected to Iceberg B9B to the west (Fig. 1). Iceberg B9B calved in 1987 in the Ross Sea and then drifted westward. In February 2010, B9B hit the Mertz glacier tongue. The calved Mertz glacier tongue, named Iceberg C28, drifted away, but Iceberg B9B arrived in Commonwealth Bay and grounded there in March 2011 (http://www.scp.byu.edu/data/iceberg/ascat/b09b.ascat). The grounding of Iceberg B9B provided favorable conditions for fast ice to grow around it, making Commonwealth Bay a permanent ice-covered region after March 2011.

On 3 December 2013, there was a large area of open water between the fast ice connected to Iceberg B9B to the east and ice floes to the west, as indicated by the arrow in Fig. 1a. But four days later, those ice floes had drifted westward (Fig. 1b) and become packed just to the east of the fast ice on 15 December 2013 (Fig. 1c). As reported in the press, Akademik Shokalskiy requested rescue on 24 December 2013, suggesting that sea ice had become further consolidated by this time. Xuelong went to the site to aid the rescue on 25 December 2013, but thick ice prevented it from getting closer than 6 nautical miles from Akademik Shokalskiy on 27 December. Xuelong reported that it was also trapped on 2 January 2014. The MODIS image on 29 December 2013 (Fig. 1d) already shows that well consolidated ice packed around those areas of fast ice including the area around Iceberg B9B, while the image on 3 January 2014 (Fig. 1e) clearly shows a sharp distinction between ice-free polynyas and areas covered by thick sea ice.

The site is close to the Adélie coastal region, which experiences some of the strongest and most persistent surface wind regimes in the world. This has been known for many years and was first reported by Sir Douglas Mawson's Australasian expedition of 1912–1913 at Cape Denison (67.01°S, 142.67°E) (‘The Home of the Blizzard', Mawson, 1915). Output from the Antarctic Mesoscale Prediction System that employs a meso-scale weather prediction model for the Antarctic region shows that the strongest wind is found around (67.5°S, 140°E), with the annual mean wind speed being approximately 20 m/s^5^. The site is slightly to the east of the region with the strongest winds.

**Figure 2:**
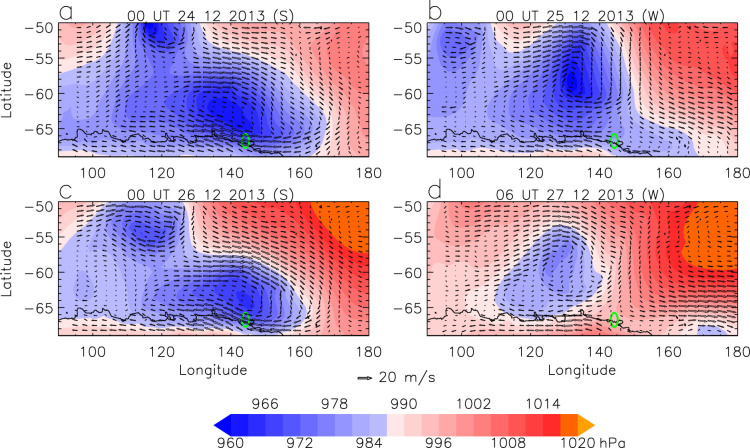
Six-hourly MSLP fields overlaid with 10-m winds at (a) 00 UT of 24 December 2013, (b) 00 UT of 25 December 2013, (c) 00 UT of 26 December 2013, (d) 06 UT of 27 December 2013. The green oval marks the site where the vessels were trapped. This figure was plotted using Interactive Data Language.

Katabatic wind events occur year round, but are greatly enhanced when cyclones move into the region^4^, typically from the west. The lows initially give strong northerly flow at locations to the east of the cyclone centre, which suppresses the katabatic flow. However, once a low has moved to the east of a site the katabatic flow is enhanced because of the strong southerly flow on the storm's western side^5^,^6^. To illustrate how the katabatic flow was affected by synoptic weather system during the event, 6-hourly mean sea level pressure (MSLP) fields and 10-m wind fields from the ECMWF operational analysis (http://www.ecmwf.int/products/data/operational_system/) for four cases were analyzed (Fig. 2). Katabatic winds became strong at 00 UT of 24 December 2013 (Fig. 2a) and at 00 UT of 26 December 2013 (Fig. 2c), and became weak at 00 UT 25 of December 2013 (Fig. 2b) and at 06 UT of 27 December 2013 (Fig. 2d); for example, at the location (66.5°S, 146°E) slightly to the east of the site, wind speed was 17.9 m/s at 00 UT of 24 December, 16.8 m/s at 00 UT of 26 December, 1.5 m/s at 00 UT of 25 December, and 0.8 m/s 06 UT of 27 December. In the coastal region, when strong wind events occurred, there were strongest winds around 140°E (Fig. 2a and 2c), which is consistent with the result in ref. 5. In such cases, since the cyclone centres were very close to 140°E or slightly to the east of 140°E, there were no strong northerly flows to suppress the katabatic winds (Fig. 2a and 2c). In contrast, when the cyclone centres shifted westward, there were relatively strong northerly flows around 140°E on the eastern side of the cyclone, thus suppressing the katabatic winds (Fig. 2b and 2d).

**Figure 3:**
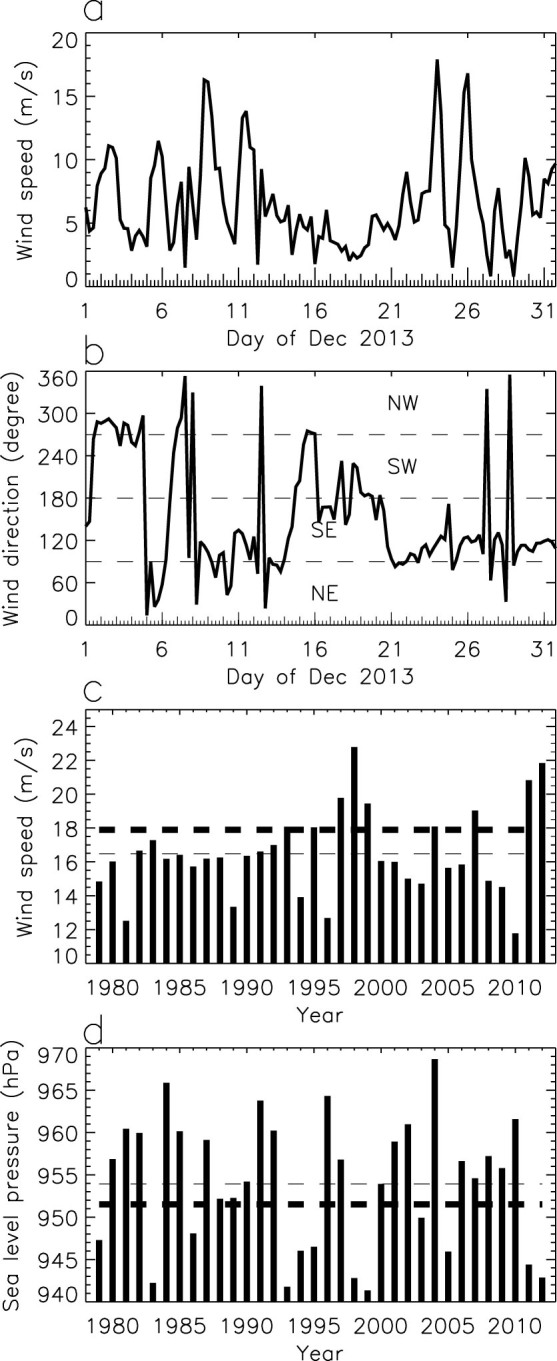
(a) 6-hourly wind speed, and (b) wind direction (defined as 0° for winds coming from the north, 90° for winds coming from the east, 180° for winds coming from the south, and 270° for winds coming from the west) at 66.5° S, 146° E for December 2013. (c) Maximum wind speed at 66.5° S, 146° E, and (d) corresponding lowest sea level pressure values of the cyclones in the sector from 70° S to 50° S and from 120° E to 150° E derived from using six-hourly data in every December of 1979 to 2012. In (c) and (d), thick dashed lines mark the values in December 2013, and thin dashed lines mark the average values over 1979–2013. This figure was plotted using Interactive Data Language.

The wind speed (Fig. 3a) and wind direction (Fig. 3b) time series at (66.5°S, 146°E) from ECMWF operational analysis 6-hourly data for December 2013 were analyzed to examine the katabatic wind changes during this month. For the wind changes immediately related to the event, the wind speed started to increase from 19 December 2013, and reached a peak value (17.9 m/s) at 00 UT 24 December 2013, corresponding to the MSLP pattern shown in Fig. 2a. The wind speed reached another peak (16.8 m/s) at 00 UT 26 December 2013 (see also Fig. 2c) after a short interruption with a minimum speed (1.5 m/s) at 00 UT 25 of December 2013 (see also Fig. 2b). These strong winds are typically southeasterly at this location, interrupted by intervals of weak winds that were typically northwesterly. Other strong wind events developed earlier in December 2013, with the peak value of wind speed (16.3 m/s) occurring at 18 UT of 8 December 2013. Note that relatively strong westerly winds developed at the beginning of December 2013 (with a wind speed of 11.1 m/s at 12 UT of 2 December 2013).

The 10-m winds and MSLP from the ERA-Interim reanalysis 6-hourly data^7^ (http://www.ecmwf.int/products/data/archive/descriptions/ei/index.html) were analyzed to identify peak wind speeds and corresponding cyclone intensities in Decembers over the period of 1979 to 2012. The strong southeasterly winds at (66.5°S, 146°E) around 23 December 2013 were not exceptional (but in the highest 29%), as stronger wind events occurred in Decembers of other 8 years derived from ERA-Interim reanalysis (Fig. 3c). Similarly, the cyclone intensity was also not the strongest (but in the deepest 37%), since deeper lows developed in Decembers of other 12 years (Fig. 3d).

Strong easterly wind events on the coast of Antarctica are often enhanced by cyclones at lower latitudes, and there is a good correlation between the strength of these events and the intensity of the cyclones^4^. The corresponding MSLP values at the cyclone centers (Fig. 3d) also have a good correlation with the peak speed values of strong winds in this particular region (Fig. 3c) for Decembers over the period of 1979 to 2013, with a correlation coefficient of 0.444 (p = 0.008).

**Figure 4:**
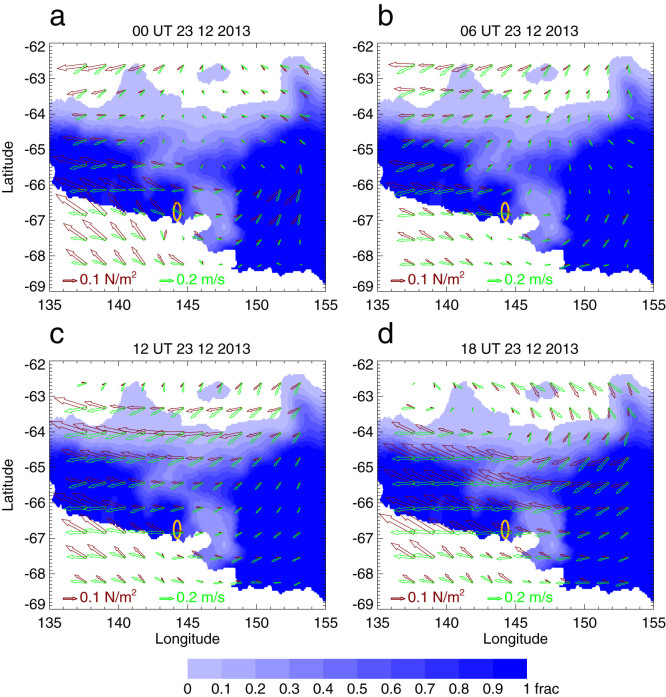
Sea ice concentration (shades of blue) overlaid by wind stress vectors (brown) and potential sea ice velocity (green) for (a) 0 UT, (b) 6 UT, (c) 12 UT, and (d) 18 UT of 23 December 2013. The yellow oval around (67° S, 144° E) marks the site where the vessels were trapped. Note that the land mask in this sea ice concentration product was not updated to reflect the calving of Mertz Glacier tongue and the grounding of Iceberg B9B. This figure was plotted using Interactive Data Language.

Sea ice concentration from the OSTIA (Operational Sea Surface Temperature and Sea Ice Analysis) dataset^8^ (http://ghrsst-pp.metoffice.com/pages/latest_analysis/ostia.html), along with wind stress derived using 10-m zonal and meridional wind components and the method in ref. 9, are employed to illustrate how sea ice around the site was moved by the wind on 23 December 2013 (Fig. 4). The OSTIA sea ice data has coarser resolution than MODIS, but it can be used to show broad scale sea ice distribution even in cloudy conditions. The use of this dataset rather than MODIS thus allows us to illustrate how broad-scale sea ice was moved by strong winds during the event.

In addition to fast ice connected with the continent at several places, there is a large amount of mobile sea ice in a broad region extending northward to about 63°S–64°S between 135°E–150°E. There were katabatic winds on the Adélie Land, with weaker winds to the east of this land. In the coastal region and around the site, katabatic winds are predominantly from the southeast, turned from the downslope direction by the Coriolis force. To the north of the site, the winds generally come from the north and are turned westward.

For ice floes that have weak internal ice stress, sea ice velocity can be estimated by the terms of air-ice and ice-water stress, and the sea surface tilt^10^. Due to the Coriolis force, sea ice rotates to the right of the imposed wind in the northern hemisphere and to the left in the southern hemisphere. (The turning angle can be as large as 90° if no other forces are taken into account.) Ice-water stress tends to reduce the turning angle, as sea ice drift is generally faster than ocean current particularly under strong wind forcing. The turning angle can be further reduced by forcing resulting from sea surface tilt in some cases. For example, there was a westward coastal current around the site, as evidenced by the westward drift of Iceberg B9B and suggested by model results^11^. This westward current is geostrophically balanced by decreasing sea surface height from south to north, which tends to cause westward sea ice drift. According to statistical studies for the Arctic region^12^, the turning angle is generally between 30°–35° for cyclone-influenced area.

To further illustrate how sea ice was moved by the wind during this event, potential sea ice drift velocity is also overlaid on sea ice concentration. Here, potential sea ice drift velocity is simply obtained by assuming a constant wind factor (the ratio of ice drift speed and overlying 10-m wind speed) of 2% and the turning angle is 30°, as ice-water stress and sea surface tilt data are not available for a quantitative estimation. The wind factor has been reported to increase with wind speed^12^,^13^,^14^; for example, the wind factor can increase from 2.3% to 2.7% with an increase of wind speed from 7 to 25 m/s for thin ice (less than 0.5 m)^13^. Here using a constant wind factor of 2% and turning angle of 30° is sufficient for a qualitative estimation of ice floe drifts.

Just to the east of the site, sea ice drifted mainly westward under the forcing of southeasterly winds, with the drift speeds, for example, of 0.15 m/s at 00, 06 and 12 UT and 0.25 m/s at 18 UT at 66.5°S, 146°E. Further to the north of Mertz polynya, there was some southward drift of sea ice. This wind-driven sea ice drift made the sea ice retreat towards the regions covered by very compact fast ice, and become much more consolidated (Fig. 1e).

The large open water area to the east of the site on 3 December 2013 (Fig. 1a) occurred after the strong westerly wind event at the beginning of December 2013 (Fig. 3a and 3b), suggesting that the strong westerly winds pushed sea ice away from the fast ice. The lack of forcing imposed by such strong westerly winds between the two strong southeasterly wind events around 8 December 2013 and 23 December 2013 led to the establishment of very thick ice to the east of the site in late December 2013, which caused this polar vessel beset event.

## Discussion

Cyclone-induced strong wind events around the site where two polar vessels were trapped were characterized by dominant southeasterly winds, as the Coriolis force turned the katabatic winds to the left when they approached the coastal region. Towards the event site, these strong southeasterly winds drove a large westward drift of ice floes, and the cyclones also drove southward ice drifts. The grounding of Iceberg B9B in Commonwealth Bay in March 2011 and the subsequent growth of fast ice around it formed a barrier for mobile ice, leading to the sea ice packing and hence extreme sea ice conditions on the eastern side of this barrier. Thus, the processes in the polar climate system on both long and short time scales caused this polar vessel beset event.

The Adélie Land coastal region of Antarctica has been identified as one of the most prominent cyclogenesis regions in the Southern Hemisphere^15^. It is thus clear that such extreme sea ice conditions must have occurred in previous years after the grounding of Iceberg B9B in March 2011, as a result of strong wind forcing on sea ice drift. This was indeed the case, as it happened at least once in the past; for example, sea ice became much more consolidated on 4 December 2012 than on 29 Nov 2012 (see MODIS images from https://earthdata.nasa.gov/labs/worldview/) after a cyclone-induced strong southeasterly wind event around 1 December 2012.

There is no doubt that similar events will happen again at this specific site with northward protruding fast ice. Future navigation should, if at all possible, avoid going to those regions where frequent strong katabatic wind events occur and with northward protruding barriers, such as fast ice, glacier tongues, or land, when large amounts of sea ice are present. Cyclone-induced strong southeasterly winds can result in extensive westward drift of sea ice, and hence have great potential to generate extreme sea ice conditions on the eastern side of these northward protruding barriers.

These results demonstrate the important role of polar cyclones or storms in the rapid creation of extreme sea ice conditions. Poleward shifts of extratropical storm tracks and intensifications of polar storm activities in a warming climate have been detected (e.g., refs. 16, 17). The intensity and frequency of such events of extreme sea ice conditions in the future will likely be affected by a global warming climate. Intensive observational and modelling studies on complicated air-sea-ice interactions in the Polar Regions are needed in order to have more comprehensive understanding of the rapid creation of extreme sea ice conditions.

## Methods

High resolution MODIS (The Moderate Resolution Imaging Spectroradiometer) daily images (https://earthdata.nasa.gov/labs/worldview/) were used to show sea ice changes around the location where the two polar vessels were trapped. Mean sea level pressure (MSLP) fields and 6-hourly 10-m wind fields from the ECMWF operational analysis (http://www.ecmwf.int/products/data/operational_system/), which has a horizontal resolution of 14 km, were analyzed to examine changes in atmospheric circulation. The peak wind speed and the associated cyclone intensity in December 2013 were compared with those in Decembers over 1979 to 2012 derived from the ERA-interim reanalysis 6-hourly data^7^ (http://www.ecmwf.int/products/data/archive/descriptions/ei/index.html). The OSTIA (Operational Sea Surface Temperature and Sea Ice Analysis) daily sea ice data (with a resolution of 5 km)^8^ (http://ghrsst-pp.metoffice.com/pages/latest_analysis/ostia.html) was employed for the purpose of illustrating cyclone-induced sea ice movements. Wind stress fields are derived using 10-m zonal and meridional wind components and the method in ref. 9. Potential sea ice drift velocity is obtained by assuming a constant wind factor (the ratio of ice drift speed and overlying 10-m wind speed) of 2% and a constant turning angle of 30°, based on the results in ref. 12.

## Author Contributions

Z.W. and J.T. conceived the study. Z.W. conducted the analysis, C.L. prepared Fig. 1, and J.T., B.S., B.L., C.L. offered useful insights during this processes. Z.W. prepared the first draft, and J.T., B.S., B.L., C.L. joined in the writing of the paper.
